# Supplementary material for: Energy release induced rockbursts based on butterfly-shaped plastic zones in roadways of coal reservoirs
Source: PLoS One. 2021 Jul 28;16(7):e0255044. doi: 10.1371/journal.pone.0255044 (PMC8318283; doi:10.1371/journal.pone.0255044)
Supplement: Attachment — Submitted filename: Review Comments to the Author.docx [file pone.0255044.s002.docx]

Review Comments to the Author
Reviewer #1: In this paper, according to the theory governing rockbursts based on butterfly-shaped plastic zones, the plane strain mechanical model for stress distribution around the holes in homogeneous elasto-plastic media was established. Based on Mohr-Coulomb yield criterion and the generalised form of Hooke’s law, the formula for elastic strain-energy density of units at a three-dimensional stress state was deduced. On this basis, the energy absorption and release in rocks surrounding a roadway during the evolution thereof in a coal reservoir with a tendency to rockbursting were quantified. This is an interesting study, but some issues should be addressed.

Response: Thanks for reviewing this manuscript. Revisions have been made based on the comments you made.
1. The author's work is very meaningful. What is the reference for the appearance of "butterfly plastic zone"? Can you describe the effect of butterfly plastic zone on rock burst in detail?

Response: The appearance of the plastic zone is defined as “butterfly-shaped” because when the confining pressure ratio η=2, the plastic zone boundary of the roadway surrounding rock is concave at the coordinate axis and protrudes on the bisector of the principal stress. At this time, there are four broadest boundaries and four smallest boundaries in the plastic zone. The plastic zone that defines the maximum boundary position is the “butterfly leaf.” When η=2.5, the boundary of the plastic zone shows prominent “butterfly” distribution characteristics. With the gradual increase in the confining pressure ratio η, the plastic zone of the surrounding rock presents an obvious uneven distribution. The plastic zone in the “butterfly leaf” is wider. When the stress ratio of the main zone rises to a particular level, the local stress field of the roadway suddenly changes due to the triggering event, causing the butterfly-shaped plastic zone in the surrounding rocks to expand in an ergodic manner. At this time, rocks are more fragile and prone to rock bursts.
2.The authors should summarize some main contributions of this paper in Section 1, and I suggest that the limitation of this work should be discussed in section 5.

Response: Contributions of this manuscript have been added at the end of Section 1, and the limitations have been supplemented in Section 5.
3. Some of the sentences are hard to understand. Grammar needs attention. The manuscript has to be carefully edited for English.

Response: Thanks for your comment. This manuscript has been edited by a native professional.
4. Note that the reference format should be standardized in the paper,and increase the citation of foreign references.

Response: The reference format has been revised, and the citation of foreign references has been increased.
